# Prevalence and diversity of gastrointestinal parasites in long-tailed macaques at Kosamphi Forest Park, Kumphawapi Monkey Garden, and Dong Ling Don Chao Pu Park, North-east Thailand

**DOI:** 10.14202/vetworld.2024.1391-1396

**Published:** 2024-06-28

**Authors:** Issarapong Phosuk, Tongjit Thanchomnang, Nattakan Puimat, Julalak Banglua, Jurairat Jongthawin

**Affiliations:** 1 Department of Public Health, Mahidol University, Amnatcharoen Campus, Amnat Charoen, Thailand; 2 Faculty of Medicine, Mahasarakham University, Maha Sarakham, Thailand; 3 Biomedical Science Research Unit, Mahasarakham University, Maha Sarakham, Thailand; 4 Protected Area Regional Office 8, Department of National Park, Wildlife and Plant Conservation, Khon Kaen, Thailand; 5 Research and Academic Services Group, Mahidol University, Amnatcharoen Campus, Amnat Charoen, Thailand

**Keywords:** Dong Ling Don Chao Pu Park, gastrointestinal parasite, Kosamphi Forest Park, Kumphawapi Monkey Garden, long-tailed macaques

## Abstract

**Background and Aim:**

These three parks in North-east Thailand, Kosamphi Forest Park, Kumphawapi Monkey Garden, and Dong Ling Don Chao Pu Park, are internationally recognized for sheltering long-tailed macaques. Overfeeding by tourists and locals significantly increases the frequency of human-macaque encounters. Being close to each other raises the chances of contracting gastrointestinal (GI) parasites. This study was designed to estimate the prevalence and range of GI parasite infections in long-tailed macaques among the three major natural habitats.

**Materials and Methods:**

Three hundred fecal samples were collected from long-tailed macaques, with 100 samples from each of the three study sites. The samples underwent Formalin-ethyl acetate concentration technique examination. Parasites were identified based on their morphology and size as observed under a light microscope.

**Results:**

About 66.67% of the population had GI parasitic infection. *Balantidium coli* had the highest occurrence (41.66%), whereas *Strongyloides* spp. (24.33%), *Trichuris* spp. (18.33%), *Entamoeba coli* (10.33%), and *Endolimax nana* (2.33%) followed next in prevalence. A higher rate of single infections (41%) was reported compared to mixed infections (25.66%). At Dong Ling Don Chao Pu Park, the prevalence rate of *B. coli* in long-tailed macaques was 70%, markedly higher than those reported at the other two study sites. In these areas, the first known case of *B. coli* infection occurred in long-tailed macaques. In the Kumphawapi Monkey Garden, the prevalence of *Strongyloides* spp. and *Trichuris* spp. infections was significantly greater (45% and 28%, respectively) compared to the other two areas.

**Conclusion:**

In northeast Thailand, long-tailed macaques are predominantly infected with *B. coli*, causing GI protozoal infections. In this primate population of the region, *Strongyloides* and *Trichuris* species are common helminths. This study offers new knowledge on parasitic loads in Thai long-tailed macaques, essential for devising effective One Health approaches to prevent and manage zoonotic diseases.

## Introduction

The long-tailed macaque (*Macaca fascicularis*), also referred to as the crab-eating macaque or cynomolgus macaque, is the predominant species of nonhuman primates (NHPs) found throughout mainland southeast Asia and its numerous islands [1, 2]. In Thailand, they are abundant and documented at 91 sites nationwide, including notable locations such as Kosamphi Forest Park in the Kosum Phisai district of Maha Sarakham province, Kumphawapi Monkey Garden in the Kumphawapi district of Udon Thani province, and Dong Ling Don Chao Pu Park in the Phana district of Amnat Charoen province, all situated in the northeastern part of the country [3–5]. Over the past decade, macaques have shifted their habitat to temple grounds and park areas near human settlements due to altered foraging behavior caused by human intervention. These macaques may pose a risk for zoonotic transmission of animal parasites to humans. Long-tailed macaques may transmit GI parasites harmful to humans. These parasites include several species, including *Strongyloides* spp., *Trichuris* spp., Hookworm, *Ascaris* spp., *Trichostrongylus* spp., *Oesophagostomum* spp., *Enterobius* spp., *Taenia* spp., and *Hymenolepis diminuta*. *Balantidium coli*, *Entamoeba coli*, *Entamoeba histolytica*, *Endolimax nana*, *Giardia lamblia*, *Blastocystis hominis*, and *Cryptosporidium* spp. have been reported as protozoan parasites in macaques [5–15]. These GI parasites can be transmitted through a fecal-oral route or by skin penetration.

In Thailand, numerous studies have identified the major gastrointestinal (GI) parasites of long-tailed macaques as *Strongyloides* spp., *Trichuris* spp., and hookworms [5–11]. *Ascaris* spp. [5, 7–8] have low prevalence rates. *Enterobius* spp. [9] and *H. diminuta* [11] were found to have similarly low prevalence rates as *Ascaris* spp. The North-east and eastern regions of the country hold the current data on GI helminth infection in long-tailed macaques. The prevalence of GI helminths in macaques from other regions of Thailand is not well documented. More research is required to determine the extent of their occurrence nationwide. Protozoan species such as *E. coli*, *E. histolytica*, *G. lamblia*, *B. hominis*, *Cryptosporidium* spp., and *E. nana* have been detected at San Phra Kan shrine and Phra Prang Sam Yod temple in central Thailand, and Khao Sam Muk in eastern Thailand [7, 12]. *E. coli* is the most commonly found among them [7]. In northeast Thailand, unlike other regions in the country, there are no reports of protozoan infections in macaques.

In Maha Sarakham province’s Kosamphi Forest Park, Udon Thani province’s Kumphawapi Monkey Garden, and Amnat Charoen province’s Dong Ling Don Chao Pu Park lie are three popular tourist destinations. Despite several publications on GI parasites, no reports exist on protozoan infections among long-tailed macaques at Kosamphi Forest Park [5–6, 8–10]. Updating the current status of GI parasite infections in macaques at Kosamphi Forest Park is essential for improving control strategies. GI parasite prevalence in long-tailed macaques at the Kumphawapi Monkey Garden remains unknown [10]. No previous reports have documented the prevalence of GI parasites in long-tailed macaques at Dong Ling Don Chao Pu Park.

This study estimated the prevalence and diversity of GI parasite infections in long-tailed macaques from three major habitats in North-east Thailand. This study provides a unique understanding of the parasitic loads of long-tailed macaques in northeastern Thailand, crucial for devising effective One Health strategies to prevent and control zoonotic diseases.

## Materials and Methods

### Ethical approval

The Mahidol University-Institute Animal Care and Use Committee (MU-IACUC) in Nakhon Pathom, Thailand, supervised and approved this research protocol under Approval Letter Number F02-64-007. All samples were collected without causing harm to the long-tailed macaques.

### Study period and location

**Figure-1:**
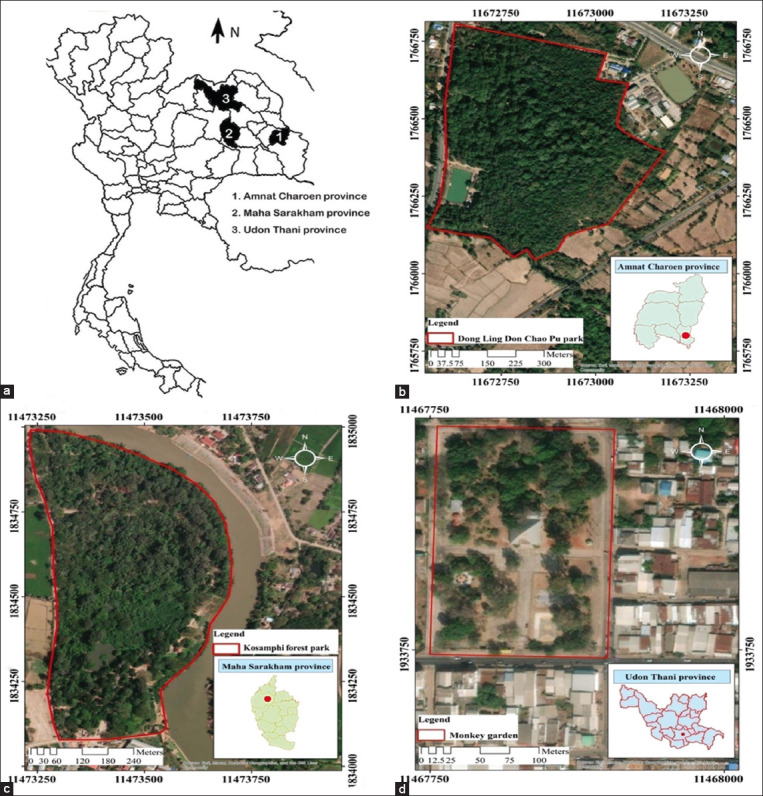
(a) The study area encompasses three provinces in northeastern Thailand. The geographical locations of sampling points include: (b) Dong Ling Don Chao Pu Park in Phana district, Amnat Charoen province; (c) Kosamphi Forest Park in Kosum Phisai district, Maha Sarakham province; (d) Kumphawapi Monkey Garden in Kumphawapi district, Udon Thani province. A red line delineates the habitat range of macaques [Source: Figure-1 (a): edited form: https://commons.wikimedia.org/wiki/File:BlankMap-Thailand-provinces.svg. Figure-1(b), (c), and (d): https://www.qgis.org/en/site/].

The study was conducted from March to April 2022 in three well-known locations that hosted long-tailed macaques in North-east Thailand. Dong Ling Don Chao Pu Park in Phana district, Amnat Charoen province; Kosamphi Forest Park in Kosum Phisai district, Maha Sarakham province; and Kumphawapi Monkey Garden in Kumphawapi district, Udon Thani province. The geographical locations of the sampling points are illustrated in Figure-1.

### Fecal sample collection

Fecal samples were obtained from 300 long-tailed macaques, with 100 samples collected from each of the following locations. Dong Ling Don Chao Pu Park, Kosamphi Forest Park, and Kumphawapi Monkey Garden. Feces were collected from the ground promptly after defecation, packed in a stool container, and kept in foam boxes during transportation to the laboratory.

### Formalin-ethyl acetate concentration technique (FECT) and fecal examination

Two grams of fecal matter was dissolved in 15 mL of 0.85% normal saline solution, and the mixture was thoroughly vortexed to dissolve the feces. The filtrate was collected in a 15 mL centrifuge tube after filtering the resulting mixture through wet gauze in a funnel. The tube was centrifuged at 500× *g* for 5 min. After discarding the supernatant, 7 mL of 10% formalin was added to the pellet. The mixture was evenly blended and let sit at room temperature for 5 min. 3 mL of ethyl acetate was added and evenly blended in. The supernatant was discarded after centrifuging the mixture once more at 500× *g* for 5 min. The cytopipette was employed to blend the residual sediment completely. 1% iodine solution was added to a drop of this mixture on a glass slide. The slide was examined under a microscope (Olympus, Japan) with a coverslip in place. Parasites were identified by their unique morphological traits, discernible under a light microscope [16, 17].

### Statistical analysis

Each GI parasite species’ prevalence was determined. Fisher’s exact test was employed to assess the statistical significance of varying parasite prevalence among long-tailed macaques at three study sites. With a confidence level of 95% and statistical significance of p < 0.05, the analyses were conducted.

## Results

Two hundred out of the 300 analyzed fecal samples (66.67%) tested positive for GI parasites. Protozoan infection prevalence was higher, with 136 (45.33%) samples, compared with helminth infection, which accounted for 106 (35.33%) samples. Infection prevalence was highest (81%) at Dong Ling Don Chao Pu Park and significantly higher than at Kosamphi Forest Park (31%) and Kumphawapi Monkey Garden (24%). In Kumphawapi Monkey Garden, the helminth infection prevalence rate was significantly higher (61%) compared to that in Dong Ling Don Chao Pu Park (29%) and Kosamphi Forest Park (16%) (Table-1).

**Table-1:** Prevalence of GI parasite infection among long-tailed macaques across three study locations.

Study locations	Number of examined	Type of GI parasite identified		

		Number of positive samples (%)		

		Protozoa	Helminth	Absence
Dong Ling Don Chao Pu Park	100	81 (81)*	29 (29)	12 (12)
Kosamphi Forest Park	100	31 (31)	16 (16)	57 (57)
Kumphawapi Monkey Garden	100	24 (24)	61 (61)**	31 (31)
Total	300	136 (45.33)^#^	106 (35.33)	100 (33.33)

* Significant difference between Dong Ling Don Chao Pu Park and each of the other two locations.

** Significant difference between Kumphawapi Monkey Garden and each of the other two locations.

# Significant difference among type of GI parasite infections. GI=Gastrointestinal

**Figure-2:**
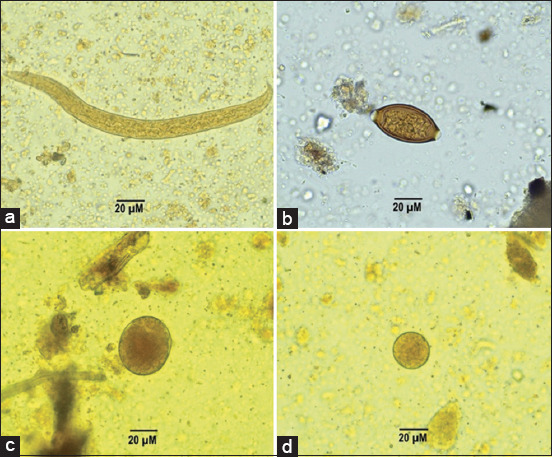
Gastrointestinal parasite identified in fecal samples of long-tailed macaques (a) *Strongyloides* spp., (b) *Trichuris* spp., (c) *Balantidium coli*, and (d) *Entamoeba coli*.

As shown in Table-2, five distinct GI parasites were identified in long-tailed macaques. The Figure-2 illustrates three protozoa species; *B. coli*, *E. nana*, and *E. coli*, as well as two helminth species, *Strongyloides* spp. and *Trichuris* spp. The prevalence rates were highest for *B. coli* (41.66%), followed by *Strongyloides* spp. (24.33%), *Trichuris* spp. (18.33%), *E. coli* (10.33%), and *E. nana* (2.33%). In Dong Ling Don Chao Pu Park, the *B. coli* prevalence rate was markedly higher (70%) than in Kumphawapi Monkey Garden (24%) and Kosamphi Forest Park (31%). In the Kumphawapi Monkey Garden, the prevalence of *Strongyloides* spp. (45%) and *Trichuris* spp. (28%) infections was notably higher than in the other two monkey sites. In this research, single infections occurred more frequently than mixed infections. About 41% had the highest infection status for single infections, while double, triple, and multiple infections accounted for 21%, 4.33%, and 0.33%, respectively (Table-3).

**Table-2:** Prevalence of GI parasite species identified among long-tailed macaques across three study locations.

Study locations	Number of examined	Species of GI parasite identified				

		Number of positive samples (%)				

		*Trichuris* spp.	*Strongyloides* spp.	*Balantidium coli*	*Entamoeba coli*	*Endolimax nana*
Dong Ling Don Chao Pu Park	100	17 (17)	20 (20)	70 (70)*	28 (28)	7 (7)
Kosamphi Forest Park	100	10 (10)	8 (8)	31 (31)	0 (0.0)	0 (0.0)
Kumphawapi Monkey Garden	100	28 (28)^#^	45 (45)**	24 (24)	3 (3)	0 (0.0)
Total	300	55 (18.33)	73 (24.33)	125 (41.66)	31 (10.33)	7 (2.33)

* Significant difference between Dong Ling Don Chao Pu Park and each of the other two locations.

** Significant difference between Kumphawapi Monkey Garden and each of the other two locations.

# Significant difference between Kumphawapi Monkey Garden and Kosamphi Forest Park. GI=Gastrointestinal

**Table-3:** Infection status of GI parasite among long-tailed macaques across three study locations.

Study locations	Number of examined	Infection status (%)			

		Single infection	2	3	≥4
Dong Ling Don Chao Pu Park	100	44 (44)	34 (34)*	9 (9)	1 (1)
Kosamphi Forest Park	100	38 (38)	5 (5)	0 (0.0)	0 (0.0)
Kumphawapi Monkey Garden	100	41 (41)	24 (24)*	4 (4)	0 (0.0)
Total	300	123 (41)	63 (21)	13 (4.33)	1 (0.33)

* Significant difference with Kosamphi Forest Park. GI=Gastrointestinal

## Discussion

GI parasites, such as helminths and protozoans, have been extensively documented in wild animals [18], and are particularly prevalent among NHPs [19–23]. In this study, the long-tailed macaques in Thailand displayed a prevalence rate of 66.67% (200/300) for GI parasites. In one Thai study [7], a prevalence of 84.1% was reported, while another study in a different location [6] found a prevalence of 62.69%. In Baluran National Park, East Java, Indonesia, and Barangay Sumile, Butuan City, Philippines, studies reported GI parasite prevalences of 80.97% [13] and 100% [24], respectively.

At the three study sites, protozoan infections occurred more frequently (45.33%) than helminthic infections (35.33%). In Dong Ling Don Chao Pu Park, the highest prevalence of protozoal infection (81%) was observed, while the prevalence of helminth infection was 29%. About 70% of protozoa in this study were *B. coli*, 28% were *E. coli*, and 7% were *E. nana*. Our study corroborates the 84.7% prevalence of *B. coli* in long-tailed macaques from non-human primate breeding facilities in Rio de Janeiro, Brazil [25]. However, our findings contrast with those of a previous study in eastern Thailand, where a high prevalence of protozoa in long-tailed macaques (73%) was documented; notably, *E. coli* was identified as the predominant species in that study, with a prevalence rate of 58.7%, whereas *B. coli* was not observed [7]. Similarly, a study conducted in Baluran National Park, Situbondo, East Java, Indonesia, reported that the highest prevalence of protozoa infection in such macaques was 89%, with *Entamoeba* spp. also identified as the predominant protozoa species (53%), whereas *B. coli* was not detected [13]. In Barangay Sumile, Butuan City, Philippines, *Eimeria* spp. infections were the most common, affecting 76.47% of long-tailed macaques [24]. In northeast Thailand, long-tailed macaques were infected predominantly with *B. coli* among GI protozoa. *B. coli* is known to parasitize various animals, including swine, ostriches, humans, and NHPs, thus presenting a potential for zoonotic transmission [26, 27]. Asymptomatic *B. coli* infections are common, but individuals with pre-existing health issues can suffer prolonged diarrhea, abdominal discomfort, and in rare cases, bowel perforation [28]. 20% of long-tailed macaques at Dong Ling Don Chao Pu Park were infected with *Strongyloides* spp., while 17% carried *Trichuris* spp. infection.

About 61% of cases at the Kumphawapi Monkey Garden had helminth infections, compared to 24% with protozoa infections. In this location, the most common helminth identified was *Strongyloides* spp. (45%), matching the 46.26% documented by Thanchonang *et al*. [10]. The first occurrences of *Trichuris* spp. and *B. coli* were noted with prevalence rates of 28% and 24%, respectively.

About 16% of long-tailed macaques in Kosamphi Forest Park had helminth infections. About 10% and 8% of the identified helminths were accounted for *Trichuris* spp. and *Strongyloides* spp., respectively. In contrast to the two recent studies [8, 9], our findings suggest a lower prevalence and a reduced number of GI parasite species. Damrongsukij *et al*. [8] found that 35.11% of the population had parasite infections, among which *Trichuris* spp. accounted for 22.90%. The prevalence rates for *Strongyloides* spp., hookworm, and *Ascaris* spp. were 15.27%, 4.58%, and 1.53%, respectively. 31% of the population in this area was reported to have parasite infections, with *Strongyloides* species and *Trichuris* species being the predominant types [9]. This study reveals a 31% prevalence of *B. coli* infection in long-tailed macaques, representing the initial documented instance in this region.

These findings underscore long-tailed macaques as critical reservoirs for GI parasites capable of transmitting to humans. Therefore, controlling and preventing the spread of these parasitic diseases among macaques and their transmission to humans is crucial. Effective control, prevention, or eradication of zoonotic diseases nowadays relies on a One Health approach - a synergistic strategy involving experts from human health, animal health, and environmental health sectors [29]. By implementing long-term preventive measures, this approach can mitigate the incidence of contagious zoonotic diseases in the future.

## Conclusion

The long-tailed macaque habitat in northeast Thailand predominantly hosts *B. coli*, which causes GI protozoal infections. These macaques in this region were predominantly infected with *Strongyloides* and *Trichuris* species. In the surveyed locations, five zoonotic GI parasites; *Strongyloides* spp., *Trichuris* spp., *B. coli*, *E. coli*, and *E. nana* were present in macaques and pose a risk for parasitic infections among locals and tourists. To prevent the spread of parasitic diseases in macaques, we must implement prevention and control programs, enhance sanitation, and educate the public. Effective management of public health issues requires collaboration among public health authorities, physicians, veterinarians, park staff, and environmental health personnel. The need for research on GI parasites in nearby human communities persists.

## Authors’ Contributions

IP and JJ: Devised and designed the experiment, conducted data analysis, interpreted the results, and contributed to drafting and revising the manuscript. IP, NP, and JB: Performed fieldwork and laboratory experiments. TT: Supervised the laboratory work and data analysis. All authors have read, reviewed, and approved the final manuscript.
